# Tuberculosis and Other Airborne Microbes in Occupational Health and Safety

**DOI:** 10.3390/ijerph17197088

**Published:** 2020-09-28

**Authors:** Esther Vaquero-Álvarez, Antonio Cubero-Atienza, Pilar Ruiz-Martínez, Manuel Vaquero-Abellán, María Dolores Redel-Macías, Pilar Aparicio-Martínez

**Affiliations:** 1 SRH Kliniken Landkreis Sigmaringen, Hohenzollernstraße 40, 72488 Sigmaringen, Germany; esther.vaquero@srh.de; 2 Departamento Ingeniería Rural, Ed Leonardo da Vinci, Campus de Rabanales, Universidad de Córdoba, 14071 Córdoba, Spain; ajcubero@uco.es (A.C.-A.); ig1remam@uco.es (M.D.R.-M.); 3 GC24 Clinical and Molecular Microbiology, Instituto Maimónides, Facultad Medicina y Enfermería, Campus de Menéndez Pidal, Universidad de Córdoba, 14071 Córdoba, Spain; mi1rumap@uco.es; 4 GC12 Clinical and Epidemiological Research in Primary Care, Instituto Maimónides, Campus de Menéndez Pidal, Universidad de Córdoba, 14071 Córdoba, Spain; en1vaabm@uco.es; 5 Departamento de Enfermería, Fisioterapia y Farmacología, Universidad de Córdoba, Campus de Menéndez Pidal, 14071 Córdoba, Spain

**Keywords:** tuberculosis, laboratories, work environment

## Abstract

Airborne pathogens and non-malignant infectious diseases such as tuberculosis are highly contagious and can have severe effects on healthcare workers. The symptoms of these diseases take time to manifest, which can prevent workers from noticing that they have been exposed until symptoms appear. The current paper sought to assess the occupational safety and preventative measures taken in laboratories in Spain, and to compare these measures with those reported by other studies worldwide. A cross-sectional study of workers (35–50 years old) was conducted using a web survey (*N* = 30), and a bibliometric analysis was carried out in the Scopus database (92 documents were selected). The occupational safety and health measures were inadequate, according to the opinions of the workers. The training (*p* < 0.01), the amount of work (*p* < 0.05), and how the workers followed their protocols (*p* < 0.001) were linked to incidents and exposure to airborne pathogens. The most significant previous publication was a report (848 citations) stating that the previous variables linked to exposure are vital for prevention. Most works focused on countries like the U.S.A. (*p* = 0.009) were reviews, with a limited number of studies focused on occupational safety.

## 1. Introduction

Since the 18th century, when biological agents first came to be identified and defined, different countries have developed methods to prevent and control outbreaks [1]. The first efforts to control environmental factors, such as production, appeared in England during the Industrial Revolution [2]. During this period, the concept of occupational safety and health (O.S.H.) was first defined [3]. Subsequently, countries started to develop methods to control pathogens among their populations [4], such as Spain’s attempts in the early 20th century to control the Spanish flu [5]. Nevertheless, only when the World Health Organization (WHO) highlighted the need for a series of pathogen-control measures to ensure the health of the working population in 1985 were such methods included and implemented in work environments [6].

In the European Union, a Council Directive called the 89/391/EEC, also known as Framework Directive, approved an occupational safety directive for the first time in 1989, focusing on improving measures to guarantee the safety and health of workers. This directive established the fundamental principles for occupational safety described a century earlier [7,8]. However, it was not until 2004 that this directive proved that the involvement of standard legislation and the government in health and safety at work has a positive influence [8,9]. Since then, public administrations have created policies and structures to ensure the prevention of risks and promote and improve working conditions [10,11]. In this sense, maintaining workers’ health has continued to be a crucial element in managing the public health of the population. However, each government has different ways of addressing the health of its workers. Governments must understand their workers and the effects of their work on their health [12,13].

One work environment that provides greater threats to its workers than other environments is health centers. Healthcare workers are exposed to many different biological pathogens, including human immunodeficiency virus, hepatitis B virus, and tuberculosis [1,14,15]. In this population, the rate of accidents and diseases related to work is around 3.2% [2]. The transmission of these agents among health professionals depends on a series of factors, the most important of which are the type of activity carried out by the worker and the effectiveness of the preventive interventions carried out [16,17]. Airborne pathogens and associated chronic respiratory diseases such as tuberculosis are highly contagious and can have severe effects on the health of workers [18]. Moreover, the symptoms of these diseases take time to manifest in the airways, which can prevent workers from realizing they have been exposed after symptoms begin [19,20,21]. For tuberculosis, which is caused by *Mycobacterium tuberculosis* (a Level 3 organism based on the biological risk it represents), public concern is based on the prevalence of the general population and healthcare workers that suffer from the disease [22]. In a report of WHO, it was estimated in 2015 that up to 2 billion people around the world suffer from a latent state of tuberculosis [23], which remains concerningly prevalent in low-risk countries, such as Italy [24]. In Italy in 2015, 2.1% of healthcare workers were diagnosed with latent tuberculosis infections [25]. Similar results were found in a previous systematic review which detailed that 2.9% of healthcare workers in low-incidence countries had latent tuberculosis [26].

Many factors may contribute to accidental exposure to a biological agent, although the main factors are still a lack of experience, skills, or knowledge in handling materials, and anxiety, fatigue, and a lack of care for oneself or other professionals [27]. For tuberculosis, the lack of knowledge about its transmission, the relevant preventive and biosafety measures, and the diagnosis of the disease seems to play an important role [25]. Moreover, the current situation with the new pandemic has highlighted the lack of professional and personal protective equipment (PPE) and adequate training provided in hospitals, which could have a major impact on the prevention of airborne pathogens [28]. Moreover, the latest studies have highlighted the need to create guidelines and training programs for undergraduate students and health professionals, especially for tuberculosis and other airborne pathogens [29]. In this sense, different studies have highlighted the importance of ensuring that healthcare workers receive training and have control measures in place, although these activities are difficult to implement [25,30,31].

Laboratories and research centers where diagnostic tests are carried out carry an inherent risk for their workers, who are commonly exposed to different pathogens, including tuberculosis. Nevertheless, most studies have focused on healthcare workers or students (mainly doctors and nurses) and based on contact with patients [26]. For laboratories and research centers, most available data relate to the early nineties, reporting a prevalence of around 7.8% in the United States or 6.7 clinical laboratory technicians out of 100,000 [32]. Other studies carried out in Korea stated that the risk of contracting tuberculosis in a laboratory is 1.4 percentage higher for microscopy technicians and 7.8 for culture/defense and sciences technicians compared to non-laboratory workers [33]. Different studies have also demonstrated that laboratories are vital for the follow-up and treatment of tuberculosis [34], with updates to their protocols and improvements in access to (and training for) PPE being fundamental [35,36]. Based on these previous observations, the current paper primarily sought to determine the conditions in laboratories related to occupational safety and preventative measures, mainly in Spain. The secondary objective was to analyze the differences between the current study’s results and those of other studies worldwide regarding occupational safety among healthcare professionals.

## 2. Materials and Methods

### 2.1. Survey Data Collection and Handling

A cross-sectional study using a reference population of workers aged 35 to 50 years old from different institutions (mainly Spanish centers) was conducted (Table 1). Most workers were from Europe (86.7%), being most participants from Spain (76.6%), followed by South American workers (13.3%). This study was carried out using a survey completed via the web, which included informed consent. The present study analyzed the preventative measures in microbiological laboratories, focusing mainly on airborne pathogens such as mycobacteria.

This study was implemented using a questionnaire previously validated by the Mycobacteria Reference Center. This questionnaire is available in English on the University of Córdoba website (http://www.uco.es/users/jcheca/index.php?go=mva/registro.html), and was subsequently translated into Spanish and German. To work online with the translated versions, the translations were transformed into online surveys using the Google Forms application (https://docs.google.com/forms/d/1eB1lUFGV37_S1e_0oXrdH4QvwUMQbLVoZzlI_B356j4/edit). The survey was divided into seven sections: information about the study, laboratory data, work volume, and worker training. All necessary information was given to workers in order to gather personal data and information related to safety at work. This questionnaire focused on laboratory data, including the type of laboratory, the techniques carried out in those centers, and whether the centers carried out any research. The work volume was assessed based on the number of samples, the time taken to culture media, the use of tools by the workers (such as the use of microscopes, cleaning, and disinfection of the working areas), and how the workers followed the relevant protocols (cleaning, eating, clothing, etc.). The training assessment focused on the initial training and ongoing training given to workers related to occupational safety and procedures in the work environment. Assessment was also based on whether the workers were informed of occupational measures, such as providing updates to protocols for biological accidents, as well as occupational diseases. The safety at work section focused on the work environment, the availability of personal protective equipment (PPE), the frequency of cleaning and disinfection carried out by cleaners and/or professional cleaning services, and the prophylaxes available to the workers.

The research project was approved by the Research Ethics Committee (Code 288, Reference 4258). The data to be processed were extracted from the answers given to the questions posed in the surveys. Participants were notified of the survey by email after voluntarily agreeing to the collection of their data.

The data collection was carried out during June 2019. The participants received an email in which they were informed about the survey’s objectives, the time allowed to complete the survey (10 min), the voluntary nature of the survey, and the possibility of not completing the survey. This survey also included a section in which the participants had to give their consent prior to completing the survey. The inclusion criteria were centers focused on the diagnosis of pulmonary diseases, both in hospitals and independent research centers, workers with a contract with the center and workers’ contact with airborne pathogens. The exclusion criteria were focused on students, non-contract worker, e.g., practices in laboratories or young predoctoral researches, and missing data. The participants who did not complete different parts of the survey did not meet the inclusion criteria and were excluded.

The independent variables included information on the study, laboratory data, work volume, training, information given to workers, and job security. The dependent variable of this study was the incidence of accidents. The data (*N* = 30) were analyzed using descriptive statistics and the relationships of the qualitative variables. Initially, data normalization was examined using the Shapiro-Wilk test. The results showed that the sample was not normalized (*p* < 0.05), so Mann–Whitney and chi-square U tests were used.

### 2.2. Bibliographic Search

Furthermore, a bibliometric analysis was carried out using the Scopus database. The bibliometric analysis was based on different Medical Subject Heading (MeSH) terms considering the study’s objective (Table 2). Additionally, the term “occupational safety” was included in the search to extract more data, although it was related to the MeSH term “occupational health”. The Boolean operators used were “OR” and “AND”, and the fields were “title”, “abstract”, and “keywords”. After obtaining all the data, SPSS program version 24 (IBM Corporation, Armonk, NY, USA), E.P.I.D.A.T. version. 4.2. (Servicio de Epidemioloxiía de la Dirección Xeral de Saúde Pública del Servicio Galego de Saúde (SERGAS), Galicia, Spain) and Excel version 17 (Microsoft Corporation, Redmond, Washington, USA) were used to analyze the information.

The bibliometric analysis was carried out in June 2020 using the following search: (TITLE ({Tuberculosis}) OR ABS ({Tuberculosis}) OR KEY({Tuberculosis}) OR TITLE ({Air Microbiology}) OR ABS ({Air Microbiology}) OR KEY ({Air Microbiology})) AND (TITLE ({occupational health}) OR ABS ({occupational health}) OR KEY ({occupational health}) OR TITLE ({occupational safety}) OR ABS ({occupational safety}) OR KEY ({occupational safety})). The exclusion criteria were articles focused on patients, or on workers that do not come in contact with airborne pathogens or whose work is not carried out in a healthcare environment. Selected studies were those related to healthcare workers and measures regarding occupational safety, with high relevance given to laboratories.

**Figure 1:**
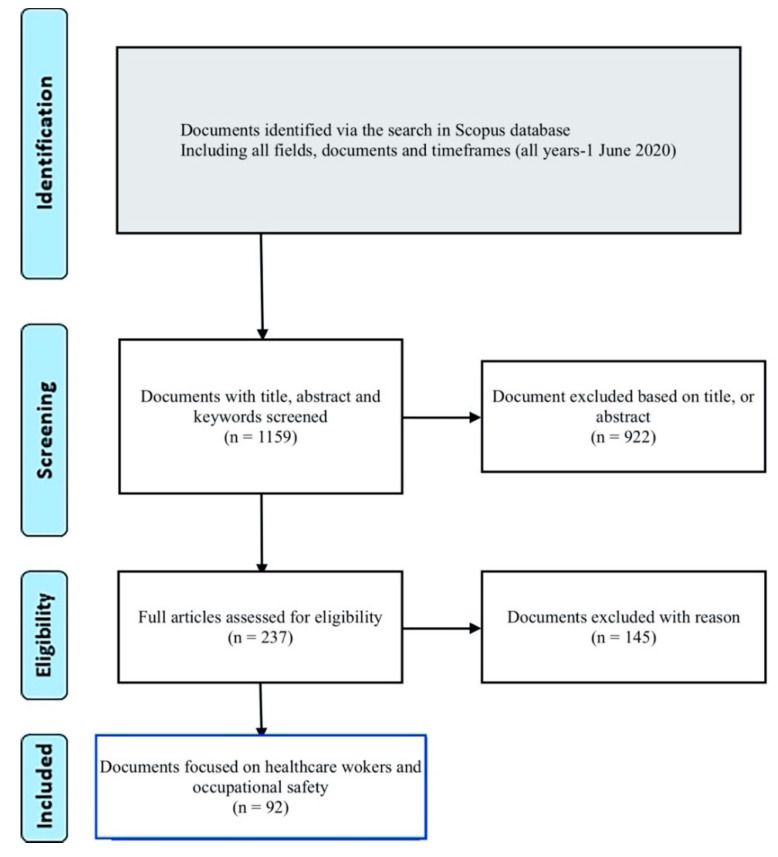
Flow diagram for the selection of articles for bibliometric analysis based on the topic and population.

In the first phase, 1159 documents published before 1 June 2020 were identified, focusing on workers, occupational safety, and airborne microbes. In the second phase, screening, 922 studies were excluded based on their titles, abstracts, and keywords. In the third phase, eligibility, exclusions were made based on the timeframe (all previous years up to the last full year (2019)), respiratory diseases related to dust or working conditions, and other work environments, such as coal mines, with 145 documents excluded (Figure 1). For quantitative analysis of the 92 documents, the Mann–Whitney, chi-square U, and Kruskal–Wallis tests were used, based on the Kolmogorov–Wilkins test (*p* < 0.05).

## 3. Results

### 3.1. Results of the Survey

The observational study’s initial analysis, based on healthcare workers in laboratories, showed that 23.3% of the participants came from international centers, while 76.7% worked in Spanish centers. In total, 13.3% of the international reference centers were from Europe (Belgium, Sweden, Bulgaria, and Latvia), and 10% were from North and South America (Mexico, Colombia, and the Dominican Republic).

The level of access that professionals have to PPE for their observational studies was analyzed. The results showed that most of the workers did not have all the necessary PPE, such as filtering facepiece (FFP) 1, 2, or 3 masks. It was also observed that international reference centers seemed to have less PPE available (Table 3). Most healthcare workers in laboratories indicated that there was not sufficient PPE to carry out their work, although the workplace measures were safe and secure (Table 4). In Spain, the percentage of workers who considered their work environments to be insufficient was 18.2% in local centers, 100% in regional centers, 57.1% in reference centers, and 100% in research centers. Moreover, in work environments where tasks related to airborne pathogens are carried out, the same percentages indicated that the available work spaces were unsafe for these tasks.

These workers also indicated how often they and other workers neglected to follow technical safety measures, such as going out in their work clothes, following the procedures for working with Mycobacterium tuberculosis, eating or smoking next to one’s workspace, etc. (Table 4).

In terms of training, 100% of the international and national workers described having adequate training. The descriptive analysis showed how 100% of the international centers carried out training regularly as ongoing training. However, at the Spanish level, the results showed that 57.1% of the reference centers’ workers did not receive this training regularly. When asked whether they were provided information on preventative measures, 28.6% of international workers in the reference centers considered themselves to have no information. However, at the national level, these values were more favorable for local centers: 18.2% of the workers answered that they had no information compared to 81.8% who considered that they did have such information. In contrast, in the regional centers focused on research, 100% of the workers answered negatively, while for the national reference centers, the distribution of responses was more balanced: 57.1% considered themselves to not have this information, while 42.9% answered that they had received this information.

The number of incidents in the last three years and occupational diseases were analyzed by center. The results showed that at the international level, 14.3% of incidents were reported among the reference center workers, although no worker developed any work-related illness. The data obtained at the national level showed a similar trend, where workers from local and regional centers did not present work-related accidents or illness, while among those surveyed at reference centers, 28.6% declared having suffered some type of accident. A nonparametric analysis was used to determine the differences between groups (those who had suffered an accident and those who had not suffered an accident). The analysis showed differences in the type of laboratory (*p* < 0.05), the numbers of samples processed per year (*p* < 0.05), the collection times of the samples (*p* < 0.01), and ongoing training for prevention (*p* < 0.05). Based on these results, correlations between the analyzed variables were determined. The analysis focused on relationships with incidents in the last three years that were shown to be linked to the type of laboratory (*p* < 0.05), the type of sample collection (*p* < 0.05), regular training for prevention (*p* < 0.001), and whether workers followed the relevant safety and hygiene protocols (*p* < 0.01).

### 3.2. Results of the Bibliographic Search

**Figure 2:**
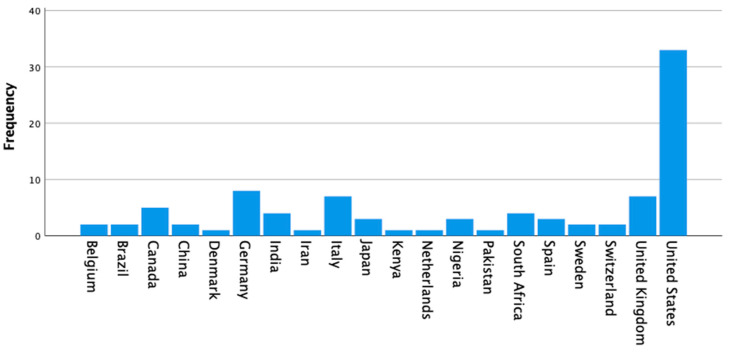
Frequency of documents per country of the bibliometric analysis with a range from one to 33 documents.

The bibliometric analysis showed that most studies focused on the United States (the USA, with 33 documents), followed by Germany (with eight documents) and the United Kingdom (the UK, with seven documents), with Spain (three documents) and other countries (Belgium and Sweden, with two documents each) poorly represented worldwide (Figure 2). Figure 2 presents the countries of origin of selected publications on the topics (occupational safety, healthcare workers, and airborne pathogens) (*N* = 92), of which most countries, such as the Netherlands and Kenya, provided only one document. The following countries with higher number of documents, including India, Italy, and South Africa, provided an average of five documents (Figure 2).

**Figure 3:**
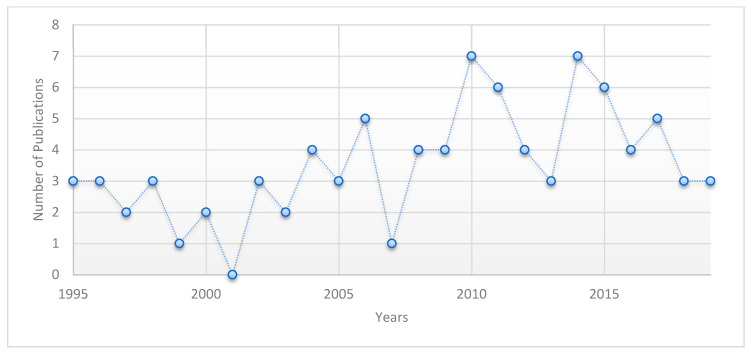
Number of publications per year of the 92 documents from the bibliometric analysis.

The number of publications per year showed significant differences between countries (*p* = 0.024), with the USA being the greatest producer of publications (*p* = 0.009). Other aspects analyzed in the previous documents included the citations, for which the cut-off point was the year 2014 (*p* = 0.037), after which the citations of the publications increased more rapidly, with an effect on the average number of publications per year (Figure 3). Figure 3 shows that many documents were published in 2014 (seven documents), although the trend from that year onward showed a decrease in the number of publications. This change (Figure 3) was similar to what was observed in 2010, when seven documents were published; that number then decreased to three documents a year by 2013. The number of citations per document differed according to the topic (*p* < 0.001), with a mean of 33 citations. The most commonly cited guidelines focused on prevention health issues among workers, with 848 citations [37]. This report highlights the importance of controlling and preventing tuberculosis for healthcare workers and the general population. For workers, the report highlights the need to control the number of workers in the testing area, provide PPE (especially respiratory protective equipment), implement a respiratory program or protocol, and provide training for healthcare workers on the use of respiratory protection.

Based on the results and recommendations, the most commonly cited observational papers were analyzed to determine the different variables and recommendations in the reports [37]. In this case, few studies included laboratories or discussed principles to prevent tuberculosis among workers (Table 5). Only one of the papers (focused on Kenya) analyzed the incidence of tuberculosis alongside one factor, which was training. The next article in terms of number of citations (eight citations) was based on factors related to prevention of tuberculosis or airborne pathogens in the United States in 1997; this study explored the relevant protocols, preventative measures, respiratory protection, and airflow regularity [38]. The results showed the improvement of the studied hospital from 1992 to 1997 (*p* < 0.01) in isolating patients with tuberculosis, assessing airflow regularity, and using PPE following the recommendations of the Centers of Disease Control and Prevention to present transmission in healthcare centers.

## 4. Discussion

The analysis of the centers from the observational study alongside the bibliometric analysis provided significant data on the prevalence of tuberculosis, the methods of its diagnosis, related working conditions, and workers’ attitudes regarding the relevant protocols.

The Spanish workers observed that there is not full compliance with preventative measures in their centers, as the protocols are not always followed. Meanwhile, the European, North and South American workers more strictly followed the relevant protocols and safety measures, such as decontamination. Another aspect that was different between Spanish and international workers was the prevalence of biological accidents and exposure to airborne pathogens, as exposure was more common among Spanish workers at local centers. These results were consistent with those of other studies, which found that centers with better control and implementation of a set of recommendations have significantly decreased exposure to airborne pathogens and fewer occurrences of biological accidents [44,45,46]. Another notable result from the observational study was the connection between a higher frequency of accidents and failure to comply with preventative regulations through ongoing training. In fact, one significant finding was the difference between Spanish and international workers regarding ongoing training. This ongoing training in prevention has been described as a necessary activity, especially for laboratory workers [47]. A previous work stated that such training education plays an essential role in identifying and circumventing outbreaks in laboratories [48]. These results match the recommendations [37] put forth by several studies [49,50], indicating the need for worker participation and ongoing training. These results agreed with those of a previous study focused on Level 3 and 4 laboratories around the world, which also indicated the need for training [51]. A previous paper focused on Spain [52] also highlighted the need to improve preventive training among Spanish workers, and how such training plays a key role in maintaining safety measures and preventing accidents. Another study focused on Russia indicated that training is lacking but essential for occupational safety, even more so under extreme or uncomfortable working conditions [53]. These previous studies agree with the reports and reviews from the health field found in the present bibliometric study [54,55,56], which indicated training as critical. However, contrary to expectations, the bibliometric study did not find a significant number of papers focused on laboratories and the measures taken by their workers. Most papers focused on determining the prevalence of tuberculosis, the proper tests to use, and comparisons between workers [39,40,41,42,43].

Another factor that was important for both Spanish and international workers was the lack of information regarding updates, protocols, or changes in the work environment. Although this lack of information was not linked to a higher risk of exposure to a biological accident, other studies have noted the importance of such information [57,58]. One paper focused on low-resource settings [59] indicated the need for information management tools to maintain constant feedback and to prevent infections among workers and patients.

Further, the manipulation of samples or cultures was linked to biological accidents and exposure to airborne pathogens in both Spanish and international centers. Samples that can generate aerosols were a significant possible risk factor for acquiring tuberculosis in the laboratory. These results coincide with those of previous studies, which determined that the longer the exposure, the higher the contagion risk. In this sense, previous studies carried out in low-incidence countries indicated how the relevant time to carry out a testing and therefore time of exposure, number of samples manipulated by workers, and type and number tests carried out, must be controlled to prevent exposure among healthcare personnel [57,60]. This risk is also related to sample concentration, the number of samples handled, and the safety measures implemented [48,61]. Another study carried out in 2016 proved that cell cultures of tuberculosis are linked to 22% of work-related contagious diseases, with laboratory technicians the most frequently affected (87%) [51].

Likewise, a large number of workers considered there to be insufficient PPE to carry out the different tasks of their laboratory safely. Among the types of PPE, HEPA filter protection masks play an important role in worker protection, especially against tuberculosis [62]. On the other hand, regarding the perceptions of worker safety and health measures at the European, North and South American levels, a small percentage of workers indicated a lack of such measures, while, in the Spanish context, researchers at the regional level and in the reference and research centers indicated a lack of security measures. This lack of security measures and PPE could be due to the decrease in public investment and purchasing of materials in the last decade, especially in the public sphere [63,64]. These results were similar to a previous review that stated how laboratories workers tough the need to improve the conditions of the work spaces available to workers to carry out their tasks [65]. The concern around such responses is based on the fact that both the CDC and the WHO have indicated the need to improve laboratory environments and employee conditions, highlighting training and the availability of adequate PPE [62].

Despite the significant results and relevance of the different variables analyzed, the bibliometric analysis proved that most studies, which were reviews or reports based on citations, have focused to date on the prevalence of tuberculosis, detection methods, and sometimes work environments. These results seem to align with those of recent studies, which were mainly focused on prevalence and risk factors, such as sex or age [66,67]. Other prior work indicated that the highest prevalence of work-related contagious illness among laboratory workers (43.4%) was related to a certain age group (women from 30 to 39 years old), which could be related to training [68]. Thus, most works focused on analyzing prevalence rather than the work environment or the worker attitudes. Indeed, the CDC described the relevant preventative factors and how to decrease such prevalence, and WHO highlighted that workers need training and constant updated, and centers should reorganize the workplaces and distribution and updated protocols every decade [34,69,70].

The current research also has limitations. The major limitation of this study is its small sample of laboratories. This research obtained data from laboratories using an observational method that limited the results to the countries that participated, the workers and email surveys invitations sent, and the timeframe used. Also, some demographic data of sample were not included, such as, position in the laboratory, level of studies and time working in the center. Another source of weakness that could have affected the bibliometric analysis is the choice of keywords. This research focused on including concise MeSH terms, which might have delimited the number of publications. Additionally, other terms such as “health work environment” or “laboratories” were not included in the search in order to limit the number of documents, as these extra terms may have resulted in a wider number of publications than desired. Finally, the Boolean operators used, “OR” and “AND”, may have included some publications which had different topics of study. However, based on the topic, population, and sample size, including a number of publications with different topics would have produced an insignificant change in the results obtained in this study.

These findings raise intriguing questions regarding the nature and extent of preventative measures taken in healthcare work environments against tuberculosis and other airborne pathogens, especially for laboratory staff. A key practical implication is the need to provide more regular training and adequate PPE for workers.

## 5. Conclusions

The aim of this paper was to assess the occupational safety and preventative measures in laboratories, in both Spain and other countries, and to determine the global differences in occupational safety among healthcare professionals.

The occupational safety and health measures in the studied laboratories were inadequate according to the opinions of the workers, both in Spain and in other European, North and South American countries. One of the most relevant findings is that biological accidents and exposure to airborne pathogens among Spanish and international workers can be linked to ongoing training, the amount of work, and how the workers followed the relevant protocols. Other factors were also noteworthy, such as the survey responses, since these data illustrated the differences and similarities between Spanish and international contexts, although the opinions of the workers were worse in Spain and were not linked to biological accidents. The results showed that in Spain, the behaviors and hygiene of workers continue to be unsafe and deficient compared to those of other European workers. These results are very interesting, since the Spanish workers expressed poorer opinions of their work environments, such as having smaller workspaces than workers from international centers.

The second major finding was that in most significant previous works, the described variables (e.g., ongoing training) were connected to a higher risk of exposure in observational studies. Another finding that emerged from the bibliometric analysis was that most works focused on countries like the USA or UK, where the incidence of tuberculosis among workers is considered low. Most of the documents from such countries were reviews, with a limited number of observational studies and studies focused on determining occupational safety and health measures. These findings suggest that to decrease the prevalence of tuberculosis and risk among workers, greater compliance with the relevant measures is needed, along with further research that focuses on whether such measures are being followed.

## Figures and Tables

**Table 1:** Demographics data of the study population.

Procedure	Sample	*N*	Frequencies
Sent out survey email invitation to one worker of each center	56 email survey email invitations sent	32 for Spain (2 per the 17 regions, except for Ceuta and Melilla) in Spain (2 per region) 6 for other European centers 6 for American centers 4 for African centers 4 for Asian centers 4 for Australia/Oceanian centers	-
Average response of the surveys sent to each worker for each center	30 surveys completed	23 in Spain 4 in other European centers 3 in American centers 0 for Asian centers 0 for Australia/Oceanian centers	71.9% in Spain 67.7% in European centers 50% in American centers 0% in Asian centers 0 in Australia/Oceanian centers
	**Workers of Each Center that Completed the Survey (*N* = 30)**		
**Variables**	**Mean (SD)**	***N***	**Frequencies**
Age	42.3(7.4)	-	-
Sex	-	20 men 10 women	66.7% men 33.3% female
Ethnic background	-	23 white (European) 3 Latino 2 African descent 2 undefined	76.6% white (European) 10% Latino 6.7% African descent 6.7% undefined
Spanish and International	-	23 Spanish workers 7 international workers	76.7% Spanish 23.3% International

Note: The minimum of the sample to achieve was 30 centers (15 Spanish and 15 International) based on previous work [32]. Additionally, for the international centers the estimation was from two to three per continent. The number achieved (*N* = 30) has been of one worker per center. A note of caution is due here since the estimations were not achieved, being higher the number of Spanish centers.

**Table 2:** MeSH terms and description.

MeSH Terms	Description	Related Terms
Tuberculosis	Any of the infectious diseases of humans and other animals caused by species of *Mycobacterium tuberculosis*	Tuberculoses Kochs Disease Koch’s Disease Koch Disease *Mycobacterium tuberculosis* Infection Infection, *Mycobacterium tuberculosis* Infections, *Mycobacterium tuberculosis* Mycobacterium *tuberculosis Infections*
Air microbiology	The presence of bacteria, viruses, and fungi in the air. This term is not restricted to pathogenic organisms.	Microbiology, Air
Occupational health	The promotion and maintenance of physical and mental health in the work environment.	Health, Occupational Industrial Hygiene Hygiene, Industrial Industrial Health Health, Industrial Safety, Occupational Occupational Safety Employee Health Health, Employee

**Table 3:** Frequency of responses of the workers.

Questions	Response	International	National			
		Reference Center	Local	Regional	Reference Center	Research Center
Is there sufficient PPE to carry out the work?	Yes	0%	9.1%	0%	0%	0%
	No	100%	90.9%	100%	100%	100%
Are there enough safety and preventative measures?	Yes	85.7%	100%	0%	71.4%	0%
	No	14.3%	0%	100%	28.6%	100%

**Table 4:** Frequency of occupational safety measures.

Question	Answer	International	National			
		Reference Center	Local	Regional	Reference Center	Research Center
Do you go outside in your work clothes?	Yes	0%	9.1%	0%	0%	0%
	No	100%	90.9%	100%	100%	100%
Do you smoke or eat close to your working area?	Yes	0%	0%	0%	0%	0%
	No	100%	90.9%	100%	100%	100%
Do you clean your workspace following protocol?	Yes	100%	100%	100%	100%	100%
	No	0%	0%	0%	0%	0%
Do you decontaminate following protocol?	Yes	100%	54.5%	100%	57.1%	100%
	No	0%	45.5%	0%	42.9%	0%
Do you wash your hands according to protocol?	Yes	100%	54.5%	100%	28.6%	100%
	No	0%	45.5%	0%	71.4%	0%

**Table 5:** The five most cited observational articles of the bibliometric analysis.

Title	Year	Country	Sample	Work environment	Variables	Results	Source	Citations
Are healthcare workers in England and Wales at an increased risk of tuberculosis? [39]	1993	United Kingdom	Healthcare workers (*N* = 119)	National Health System	Sex, ethnicity, association with other workers	Crude notification rate among healthcare workers was 11.8 per 100,000 per year	British Medical Journal (BMJ)	63
Challenges with QuantiFERON-TB Gold Assay for Large-Scale, Routine Screening of U.S. Healthcare Workers [40]	2008–2010	United States	Serial testing results of healthcare workers (*N* = 9153)	National Health System	QuantiFERON-TB Gold In-Tube test, age, sex, QFT results, including the T.B. Antigen, Nil, and Mitogen; and the test run date.	Remaining false positive (*p* < 0.001)	American Journal of Respiratory and Critical Care Medicine	61
Evaluation of Interferon-Gamma Release Assays in the Diagnosis of Recent Tuberculosis Infection in Healthcare Workers [41]	2004–2005	Spain	Testing of healthcare workers (*N* = 147)	National Health System (West)	QuantiFERON-TB GOLD In-Tube and T-SPOT. T.B. in H.C.W.s, comparing the results with a tuberculin skin test (T.S.T.)	A low frequency of B.G.G. vaccination (15.6%); the occupational degree exposure was significant when the outcome was a positive T-SPOT. T.B. result (OR = 2.03)	Plos One	30
Tuberculosis Risk Among Staff of a Large Public Hospital in Kenya [42]	2003–2005	Kenya	Healthcare workers (*N* = 4833)	National Health System	Sex, job designation, years working, household, and guidelines	The time with the patient was liked to tuberculosis (OR = 1.3) Working role (*p* = 0.07)	International Union Against Tuberculosis and Lung Disease	24
High incidence of latent tuberculous infection among South African health workers: An urgent call for action [43]	2008	Kenya	Healthcare workers (*N* = 199)	National Health System	Sociodemographic details, knowledge and risk perceptions of T.B. and L.T.B.I., and training and practice in infection control, I.G.R.A. compared with tuberculin skin test (T.S.T.)	Knowledge and infection control training and practice were associated with a 50–60% reduction in the risk of tuberculosis acquisition	International Union Against Tuberculosis and Lung Disease	23
